# Editorial: Genomic Ancestry and Biological Traits

**DOI:** 10.3389/fgene.2021.754725

**Published:** 2021-09-06

**Authors:** Fernanda Rodrigues-Soares, Fernanda S. G. Kehdy, Mateus H. Gouveia

**Affiliations:** ^1^Departamento de Patologia, Genética e Evolução, Instituto de Ciências Biológicas e Naturais, Universidade Federal do Triângulo Mineiro, Uberaba, Brazil; ^2^Laboratório de Hanseníase, Instituto Oswaldo Cruz, FIOCRUZ, Rio de Janeiro, Brazil; ^3^Center for Research on Genomics and Global Health, National Human Genome Research Institute, National Institutes of Health, Bethesda, MD, United States

**Keywords:** genomic ancestry, AdmixSim, lactase persistence, population genetics, admixture

Human genomic ancestries are proportions of an individual's genome coming from each specific ancestral population. Genomic ancestry is detectable by the difference in frequency of certain variants in the genome between ancestral populations that originated a given population and have been recurrently associated with different biological traits, including rare and common diseases such as sickle cell disease and hypertension, which are overrepresented among those with African ancestry. Especially in admixed populations (e.g., Latin Americans and African Americans) that have a variety of continental ancestral origins, population structure, and its underlying genomic ancestries might lead to spurious associations in genome-wide association studies (GWAS), if not properly accounted for. This Research Topic aims to provide additional evidence about methods to better infer genomic ancestry, its dynamics processes and its possible implications on biological traits. Yang et al. presented a new software tool (AdmixSim) to perform computer simulations of biological admixture in distinct demographic scenarios, such as multiple waves of admixture events, multiple ancestral populations, fluctuating population size, and fluctuating admixture proportions. As users are allowed to modify many parameters, it is a very flexible software that can be useful in many contexts to elucidate admixture processes and support future health and evolutionary studies. Additionally, Lin et al. showed that admixed populations can represent a powerful tool and opportunity for discovering variants associated with complex traits. They developed a mechanistic simulation framework (APRICOT) to investigate the statistical power and transferability of GWAS across a range of realistic scenarios of genotypic and ancestry-associated contributions. They demonstrated that the investigation of the genetic architecture of biological traits in admixed populations offers advantages when compared to GWAS in single-ancestral cohorts with the same sample size. These findings revealed that the study of genomic ancestry in admixed populations can improve downstream applications such as personalized medicine. In a different context, Alves et al. investigated the distribution of European haplotypes, focusing on *-13910C*>*T* (rs4988235 - *MCM6* gene), associated with lactase persistence, in 7,428 Pan-American individuals. There is no information about the consumption of dairy products in the Americas before the arrival of Europeans. In fact, previous studies suggest that most Native Americans did not digest lactose properly, and that -13910^*^T, one of the most important alleles responsible for lactose tolerance, was introduced in the Americas after European settlement. The results show a low frequency of -13910^*^T in individuals with low European ancestry, indicating a high probability of lactose intolerance and suggesting that dietary guidelines encouraging dairy product consumption may not be the most appropriate for these populations. Liu et al. explored the ancestral origin and genomic history of the Chinese Hui people. They performed a population genomics study using genome-wide data from 109 western Chinese individuals. They showed that the Hui people have a strong genomic similarity with modern and ancient North East Asians, supporting the hypothesis that the Huis arose from an admixture event involving a predominantly East Asian origin. Thus, the variety of approaches published in this topic shows how genomic ancestry studies are relevant to the scientific literature. Despite the recent efforts on understanding genomic ancestries, there is still a lack of knowledge about fine-scale population structure, admixture dynamics, and associations between subcontinental ancestries and biological traits. A comprehensive understanding of genomic ancestries will facilitate the inclusion of diverse populations in genetic studies, including admixed individuals, which are frequently excluded from genomic association analyses due to concerns over population structure.

## Author Contributions

FR-S, FK, and MG co-edited the Research Topic and co-wrote, co-edited, and co-approved the final version of the editorial for publication. All authors contributed to the article and approved the submitted version.

## Conflict of Interest

The authors declare that the research was conducted in the absence of any commercial or financial relationships that could be construed as a potential conflict of interest.

## Publisher's Note

All claims expressed in this article are solely those of the authors and do not necessarily represent those of their affiliated organizations, or those of the publisher, the editors and the reviewers. Any product that may be evaluated in this article, or claim that may be made by its manufacturer, is not guaranteed or endorsed by the publisher.

